# Effects of semaglutide on metabolism and gut microbiota in high-fat diet-induced obese mice

**DOI:** 10.3389/fphar.2025.1562896

**Published:** 2025-06-02

**Authors:** Luyan Sun, Bingqing Shang, Suyuan Lv, Guolong Liu, Qiu Wu, Yue Geng

**Affiliations:** Key Laboratory of Food Nutrition and Safety of SDNU, Provincial Key Laboratory of Animal Resistant Biology, College of Life Science, Shandong Normal University, Jinan, China

**Keywords:** obesity, semaglutide, gut microbiota, metabolomics, fecal microbiota transplantation

## Abstract

**Background:**

The purpose of this study was to explore how semaglutide, a GLP-1RA, regulates serum metabolism and gut microbiota to improve obesity in mice and whether fecal microbiota transplantation (FMT) can transmit the beneficial effects of semaglutide to recipient mice.

**Methods:**

Male C57BL/6J mice were given standard diet (ND), high-fat diet (HFD), or high-fat diet with semaglutide (SHF, 100 μg/kg). Fecal microbiota transplantation was used to transplant the fecal suspension supernatant (MT) and bacteria (FMT) from SHF group mice to antibiotic-induced pseudo-germ-free mice.

**Results:**

Results showed that semaglutide significantly reduced the body weight, body fat, FBG, and insulin levels induced by high-fat diet, and improved insulin resistance and sensitivity damage (*p* < 0.05). This was achieved by regulating the expression of genes related to lipid metabolism such as *Pparα, Pparγ, Cpt1a*, and *Pgc1α* in the liver and adipose tissue, as well as the appetite-related genes *Leptin, Agrp, Npy,* and *Pomc* in the hypothalamus. After stopping semaglutide intervention 4 weeks, the body weight of the mice rebounded significantly. Fecal microbiota transplantation could transmit the beneficial effects of semaglutide to recipient mice. Semaglutide and fecal microbiota transplantation affected metabolic pathways such as serum amino acid metabolism and pyrimidine metabolism in obese mice, and reshaped the composition and proportion of fecal gut microbiota in obese mice.

**Conclusion:**

In summary, semaglutide could inhibit food intake and improve obesity, regulate serum metabolism and the composition of gut microbiota in mice. Bacterial transplantation is key to transmitting the improvement brought about by fecal microbiota transplantation of semaglutide to recipient mice.

## Graphical Abstract

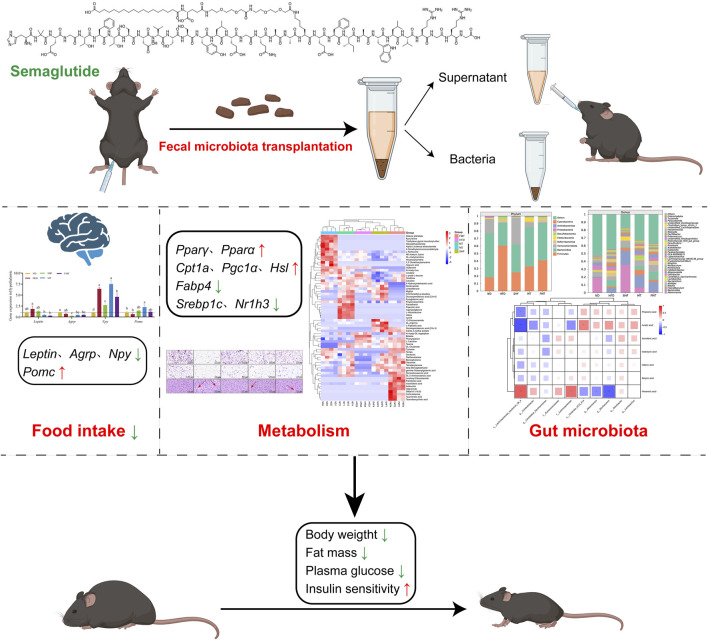

## 1 Introduction

According to a report released by the World Obesity Federation in 2023, more than 10% of adults worldwide are currently obese, and this figure is expected to rise to 17% by 2025 (Lobstein et al., 2023). Obesity is a risk factor for a range of chronic diseases, including cardiovascular diseases (Emerging Risk Factors Collaboration et al., 2011; GBD, 2015 Obesity Collaborators et al., 2017), type 1 and type 2 diabetes, non-alcoholic fatty liver disease (Canfora et al., 2019), various cancers (Lauby-Secretan et al., 2016), and osteoarthritis (Jiang et al., 2012).

Glucagon-like peptide-1 (GLP-1) is a peptide produced by the cleavage of proglucagon, mainly synthesized in the intestinal mucosal L-cells, pancreatic islet α-cells, and neurons in the nucleus of the solitary tract (Bu et al., 2024). Some gut microbiota metabolites promote the secretion of GLP-1. GLP-1 exerts an influence on the brain, intestine, and pancreas to improve host metabolism (Zeng et al., 2024). GLP-1 acts through the GLP-1 receptor (GLP-1R) expressed in many tissues. In the pancreas, GLP-1R stimulates β-cells, resulting in glucose-dependent insulin secretion (Sandoval and D'Alessio, 2015). Semaglutide, a GLP-1R agonist (GLP-1RA), has a 94% homology to endogenous GLP-1 (7–37). It extended the half life period of natural GLP-1 by modifying with amino isobutyric acid (Aib) (Deacon et al., 1998) and arginine substitutions (Lau et al., 2015) and adding a C18 fatty acid side chain. It was applied clinically as an antidiabetic drug initially, clinical studies have found that semaglutide could effectively reduces body weight in patients with diabetes (O'Neil et al., 2018; Rubino et al., 2022; Wadden et al., 2021) and also in obese patients without diabetes (Kauffman, 2022). It primarily promotes weight loss by suppressing appetite and reducing energy intake (Friedrichsen et al., 2021). Animal studies related to semaglutide have confirmed that, in addition to reducing weight, semaglutide could improves insulin resistance-induced islet damage in obese mice as well through pro-inflammatory pathways, PDX1, PPARα and PPARγ pathways (Marinho et al., 2022). It is also beneficial for the liver of obese mice via the mTORC1/AMPK pathway (Reis-Barbosa et al., 2022) and stimulates subcutaneous fat browning to alleviate inflammation and endoplasmic reticulum stress (Martins et al., 2022). From a metabolic perspective, it has been found that semaglutide could reduce obesity in mice by regulating skeletal muscle metabolism (Ren et al., 2022), however, its effects on the metabolism of other tissues have not been reported. GLP-1RAs were closely related to food intake. Upon activation of the GLP-1 receptor, the gut-brain axis could decrease the body weight by increasing the levels of GLP-1 or directly reducing food intake (Horne et al., 2020). Studies have shown that semaglutide could reduce the body weight of mice through distributed neural pathways (Gabery et al., 2020), enhancing the activity of exogenous cholecystokinin-inhibitory neurons to reduce appetite (Ghidewon et al., 2022). Research has also discovered a novel anti-obesity mechanism for liraglutide (another GLP-1RA), which improves glucose and lipid metabolism in mice and also ameliorates leptin resistance and oxidative stress in white adipose tissue (Lyu et al., 2022). From the perspective of gut microbiota, liraglutide has been found to reduce obesity-related microbial phenotypes and increase lean-related microbial phenotypes (Zhao et al., 2018). However, the effects of semaglutide on gut microbiota require further investigation.

Obesity is closely related to the gut microbiota, not only through the microbes themselves but also through their metabolites, such as short-chain fatty acids and bile acids (van der Vossen et al., 2022). Fecal microbiota transplantation is one of the five current methods targeting the gut microbiota for the treatment of obesity. It can mitigate high-fat diet-induced steatohepatitis in mice by restoring the beneficial effects of the gut microbiota (Zhou et al., 2017) and reduce obesity in both humans and mice (Ridaura et al., 2013). Clinical trials have demonstrated that transplanting the feces from lean individuals into obese individuals could significantly improves insulin sensitivity and increases butyrate-producing bacteria in the intestinal tract (Vrieze et al., 2012). Numerous animal studies have shown that interventions such as *Lactobacillus acidophilus* (Kang et al., 2022), theabrownin (Li HY. et al., 2022), anthocyanins (Liu et al., 2021), calorie-restricted diets (Wang et al., 2018; Li M. et al., 2022), and metformin (Lee et al., 2018) could utilize fecal microbiota transplantation technology to improve obesity in mice through the gut microbiota.

At present, semaglutide has an outstanding performance in improving obesity, but the effects of semaglutide on serum metabolism and gut microbiota in obese mice are still unknown. We hypothesized that semaglutide can affect the serum metabolism of mice, especially the metabolism of amino acids and lipids, and affect the gut microbiota to improve obesity. Therefore, this study explored the effects of semaglutide on the gut microbiota of obese mice induced by high-fat diet. Combined with fecal microbiota transplantation technology, by integrating metabolomics, lipid-metabolism-related genes and obesity-related indicators, the association between various indicators of mice and obesity under the intervention of semaglutide was revealed, and whether fecal microbiota transplantation technology can transmit the weight-loss effect of semaglutide was explored. It provides some new ideas for the role of semaglutide in improving obesity, reducing lipids and losing weight.

## 2 Materials and methods

### 2.1 Reagents and instruments

#### 2.1.1 Main reagents

High fat feed (60 kcal% fat) (Ready Dietech (Shenzhen) Co., Ltd., China); Semaglutide (98%, S28059), Ampicillin sodium salt (USP, S17018), Neomycin sulfate (USP, S17028) and Vancomycin HCl (USP, S17059) (Shanghai yuanye Bio-Technology Co., Ltd., China); Metronidazole tablets (H37022894, Shandong Qidu Pharmaceutical Co., Ltd., China); Triglyceride (TC) assay kit, Total cholesterol (TG) assay kit, Low-density lipoprotein cholesterol (LDL-C) assay kit, High-density lipoprotein cholesterol (HDL-C) assay kit and Insulin Assay Kit (Nanjing Jiancheng Bioengineering Institute, China); Acetonitrile (HPLC, Sinopharm Chemical Reagent Co., Ltd., China).

#### 2.1.2 Main instruments

Blood glucose tester AG-605, Blood glucose test paper (Andon Health Co., Ltd., China); Microplate Reader AMR-100T (Hangzhou Allsheng Instruments Co., Ltd. China); Ultimate 3000-Q Exactive High-Performance Liquid Chromatography (UHPLC) system (Thermo Fisher, Waltham, MA, United States).

### 2.2 Animals and experimental design

The experiment has evaluated and approved by the Experimental Animal Ethics Review Committee of Shandong Normal University (No.AEECSDNU2023011). The mice were reared in the SPF-level Experimental Animal Center of Shandong Normal University and were housed under controlled temperature (23°C ± 2°C) with an alternating 12/12 light-dark cycle (lights on at 07:00, off at 19:00). Food and water were available *ad libitum*. The whole experiment has strictly adhered to the “Administrative Regulations on Experimental Animals of China (Draft Revision for Public Comments as of 2024)” and ensured that the experimental animals meet the requirements.

#### 2.2.1 Effects of different doses of semaglutide on growth and metabolism in obese mice

**FIGURE 1:**
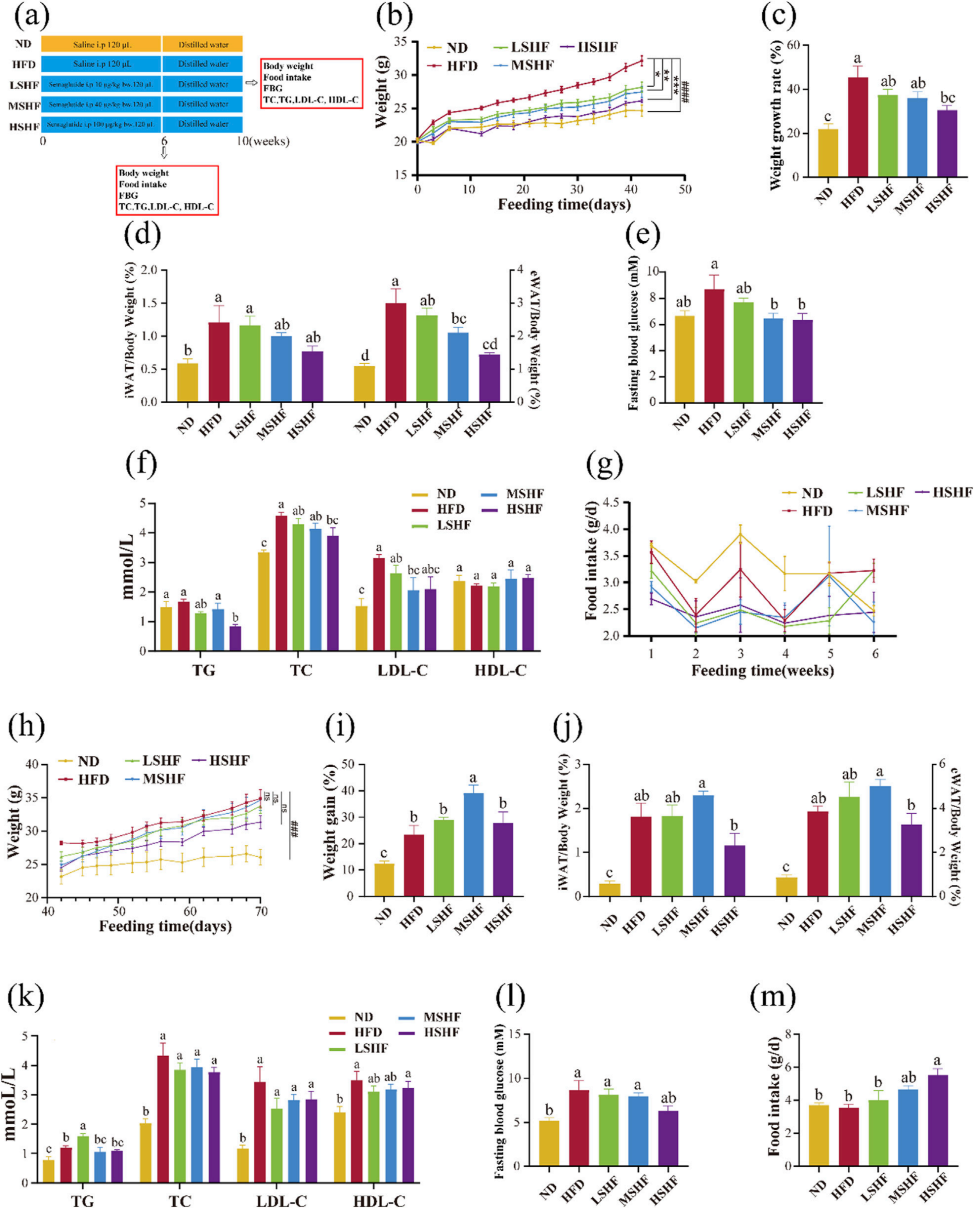
Effects of different doses of semaglutide on the growth of obese mice experimental procedure **(A)** (n = 10). Changes of body weight **(B)**, body weight growth rate **(C)**, body fat ratio **(D)**, fasting blood glucose level **(E)**, lipid profile **(F)** and food intake **(G)** in obese mice after 6 weeks of semaglutide intervention at different doses (n = 6). After a 4-week cessation of semaglutide intervention, the changes in body weight **(H)**, body weight growth rate **(I)**, body fat ratio **(J)**, lipid profile **(K)**, fasting blood glucose level **(L)** and food intake **(M)** in obese mice (n = 4). Note: ^###^ *p* < 0.0005, ^####^ *p* < 0.0001 vs. ND; *p < 0.05, **p < 0.01, ***p < 0.005, ns *p* > 0.05 vs. HFD, and significant differences are indicated by different letters on the bar graph (*p* < 0.05).

Four-week-old SPF male C57BL/6J mice were purchased from Beijing Vital River Laboratory Animal Technology Co., Ltd. [SCXK (Jing) 2021-0011]. Centralized adaptive feeding for 1 week, free drinking water, diet (common feed). After 1 week of adaptive feeding, the mice were randomly divided into five groups: normal group (ND, n = 10), obesity model group (HFD, n = 10), low-dose semaglutide group (LSHF, n = 10), medium-dose semaglutide group (MSHF, n = 10) and high-dose semaglutide group (HSHF, n = 10). During the whole experiment, the ND group were fed with standard diet, and the other mice were fed with high fat diet (60 kcal% fat) for 10 weeks. The mice were intraperitoneal injected 120 μL physiological saline every other day in the ND and HFD groups, and 10 μg/kg·bw, 40 μg/kg·bw and 100 μg/kg·bw semaglutide dissolved in physiological saline every other day in the LSHF, MSHF and HSHF group respectively for 6 weeks. Six mice in each group were euthanised after 6 weeks, the remains were kept on the same diet without any intervention, and were euthanised in the end of the 10th week. In addition, body weight, food intake and fasting blood glucose (FBG) measurements were performed weekly during the 10-week intervention. Throughout the experiment, mice were fed and watered *ad libitum* (Figure 1A).

#### 2.2.2 Effects of semaglutide and fecal microbiota transplantation on growth, metabolism and gut microbiota in obese mice

Four-week-old SPF male C57BL/6J mice were purchased from Beijing Vital River Laboratory Animal Technology Co., Ltd. [SCXK (Jing) 2021–0011]. Centralized adaptive feeding for 1 week, free drinking water, diet (normal feed), room temperature 23°C ± 2°C. After 1 week of adaptive feeding, the mice were randomly divided into three groups: normal group (ND, n = 9), obesity model group (HFD, n = 27) and semaglutide group (SHF, n = 9). During the whole experiment, the ND group were fed with standard diet, and the other mice were fed with high fat diet (60 kcal% fat) for 10 weeks. The mice were intraperitoneal injected 120 μL physiological saline in the ND and HFD groups, and 100 μg/kg·bw semaglutide dissolved in physiological saline every other day in the SHF group reapectively. After 4 weeks, the HFD group was subdivided into HFD group (n = 9), transplanted supernatant group (MT, n = 9) and transplanted bacteria group (FMT, n = 9) for fecal microbiota transplantation as follows:

Fecal Sample Collection:Take the fresh feces of mice in the SHF group, dilute them with sterile PBS at a ratio of 0.1 g/mL, then vortex, centrifuge at 800 rpm for 3 min to collect the supernatant, and remove the impurities in the fecal slurry The supernatant was centrifuged again (12,000 rpm, 1–2 min), and the supernatant (bacterial metabolites) obtained was used in the MT group. The organisms left at the bottom of the tube were resuspended again with PBS to half of the original volume, and the resuspended liquid (bacteria) was used in the FMT group.

Fecal microbiota transplantation: MT group and FMT group after the depletion of gut microbiota by antibiotic cocktails (drinking water containing 1 g/L ampicillin, neomycin and metronidazole, and 0.5 g/L vancomycin daily for 2 weeks), were gavaged respectively every other day with freshly-prepared stool suspensions 200 μL for 4 weeks.

**FIGURE 2:**
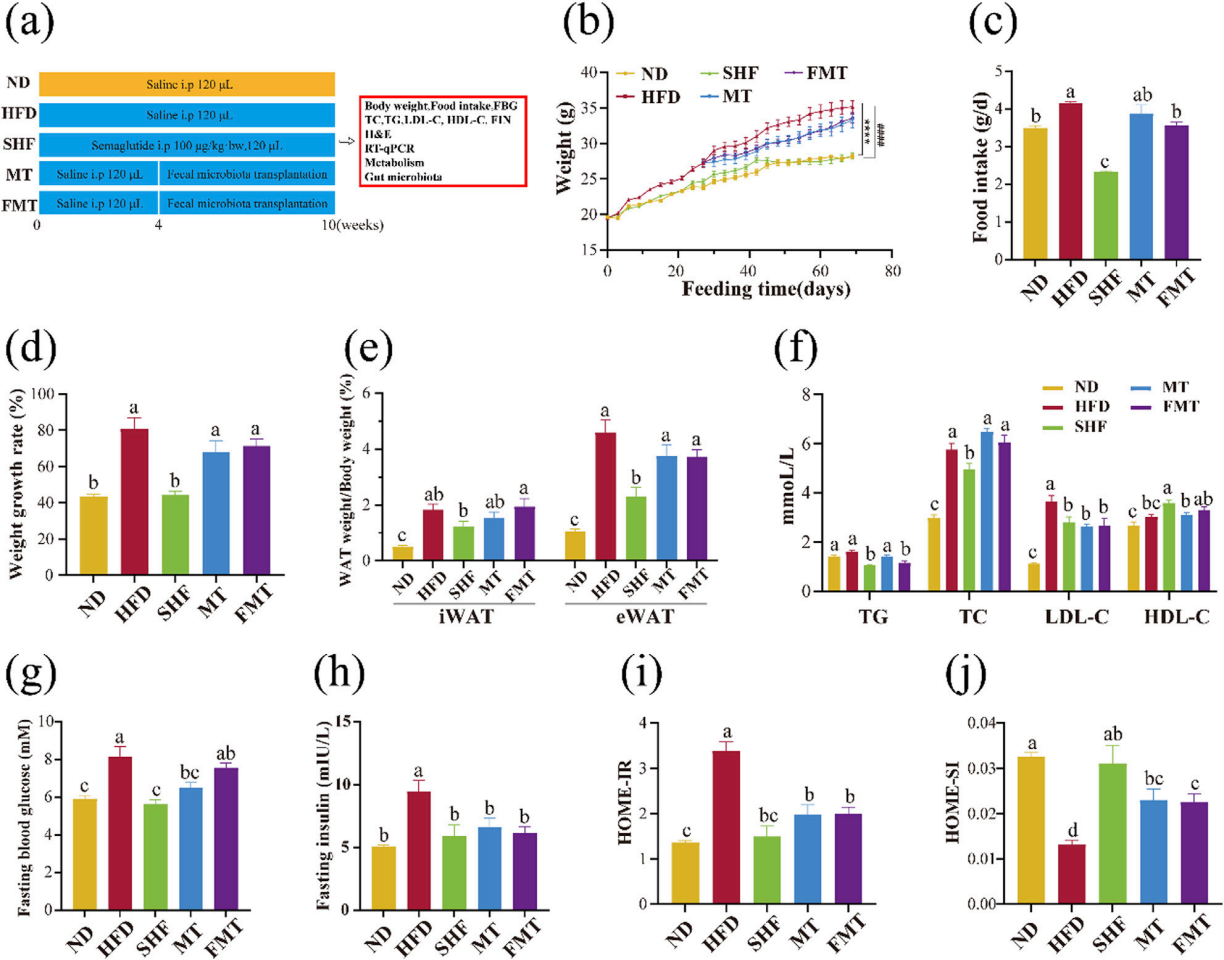
Effect of semaglutide and fecal microbiota transplantation on the growth of obese mice (n = 9) Experimental procedure **(A)**. body weight change **(B)**, food intake **(C)**, body weight growth rate **(D)**, body fat ratio **(E)**, lipid profile **(F)**, fasting blood glucose level **(G)**, fasting insulin level **(H)**, HOMA-IR **(I)** and HOMA-IS **(J)** Note: ^####^ *p* < 0.0001 vs. ND; ****p < 0.0001 vs. HFD, and significant differences are indicated by different letters on the bar graph (*p* < 0.05).

The whole operation should be carried out in a sterile and low temperature environment. All mice in each group were euthanised after experiment and body weight`food intake and FBG measurements were performed weekly during the 10-week intervention (Figure 2A).

### 2.3 TC, TG, LDL-C, HDL-C, fasting insulin measurements

Serum TC, TG, LDL-C, HDL-C and fasting insulin (FIN) levels were measured in mice using the Assay Kit form Nanjing Jiancheng Bioengineering Institute.

### 2.4 Adipose tissue and liver morphology

The mouse inguinal white adipose tissue (iWAT), epididymis white adipose tissue (eWAT) and liver were fixed with 4% paraformaldehyde for 24 h, then paraffin sections were dewaxed to water, stain with hematoxylin for 3–5 min followed by eosin for 5 min. Images were taken by a Nikon Eclipse E100 microscope. The histological effects of different interventions on mice were evaluated by measuring the average size of adipocytes in adipose tissue and the average area of lipid droplets in liver tissue using ImageJ software (NIH, Bethesda, MD, United States). Five fields of view (20 x) of adipose tissue sections stained and five fields of view (40 x) of liver tissue sections stained were selected for measurement. The details on image analysis-based quantification of steatosis and adipocytes and ImageJ macro code are provided in the Supplementary Material, along with representative example images (see Supplementary Figure S1).

### 2.5 Tissues mRNA gene expression by real-time qPCR

iWAT, eWAT, liver and hypothalamus gene expression was analyzed by RT-qPCR. We asked Wuhan Servicebio Technology CO., Ltd. to conduct the experiment. The expression of target genes was normalized to expression of *Gapdh*, and shown as fold change relative to the control group based on the 2^−ΔΔCT^ method. The primer sequences were shown in Table 1. Cycling conditions as follows:① Pre-denaturation at 95°C for 30s ② Denaturation at 95°C for 15s, annealing/extension at 60°Cfor 30s and 40 cycles.③Melting curve: 65°C→95°C, fluorescence signals were collected once every 0.5°C warming. Excessive concentrations of RNA were diluted in appropriate proportions to achieve a final concentration of 200 ng/μL.

**TABLE 1:** RT-qPCR primer sequences.

Gene	S	As
*Gapdh*	CCT​CGT​CCC​GTA​GAC​AAA​ATA​G	CCT​CGT​CCC​GTA​GAC​AAA​ATA​G
*Pparα*	CAA​GGC​CTC​AGG​GTA​CCA​CTA​C	GCC​GAA​TAG​TTC​GCC​GAA​A
*Nr1h3*	CTT​TAG​GGA​TAG​GGT​TGG​AGT​CAG	AGA​CAT​AGC​GTG​CTC​CCT​TGA​T
*Srebp1c*	GAC​ATG​CTC​CAG​CTC​ATC​AAC​A	GAC​ACG​GAC​GGG​TAC​ATC​TTT​A
*Pparγ*	GGA​AGA​CCA​CTC​GCA​TTC​CTT	GTA​ATC​AGC​AAC​CAT​TGG​GTC​A
*Pgc1α*	TAT​GGA​GTG​ACA​TAG​AGT​GTG​CT	GTC​GCT​ACA​CCA​CTT​CAA​TCC
*Cpt1a*	AGA​TCA​ATC​GGA​CCC​TAG​ACA​C	CAG​CGA​GTA​GCG​CAT​AGT​CA
*Hsl*	GAT​TTA​CGC​ACG​ATG​ACA​CAG​T	ACC​TGC​AAA​GAC​ATT​AGA​CAG​C
*Fabp4*	CGA​TGA​AAT​CAC​CGC​AGA​CG	CCA​GCT​TGT​CAC​CAT​CTC​GT
*Agrp*	CGG​CCA​CGA​ACC​TCT​GTA​G	CTCATCCCCTGCCTTTGC
*Npy*	CCG​CCA​CGA​TGC​TAG​GTA​AC	CAG​CCA​GAA​TGC​CCA​AAC​AC
*Pomc*	AGG​CGA​CGG​AAG​AGA​AAA​GA	AGA​TTG​GAG​GGA​CCC​CTG​T
*Leptin*	CTG​TGG​CTT​TGG​TCC​TAT​C	TGA​TGA​GGG​TTT​TGG​TGT​CA

### 2.6 Serums UHPLC-QE-MS analysis

Mouse serum samples were treated with the liquid mixture (serum:water:acetonitrile = 1:2:4.5) and vortex shaken for 60 s (Luo et al., 2024). The mixed solution was centrifuged at 12,000 rpm for 10 min at 4°C. The supernatant was filtered with a 0.22 μm membrane (ANPEL, Shanghai, China) into labeled autosampler vials using disposable syringes (Shinva ANDE, Zibo, China) and stored at 4°C for later analysis.

A UHPLC-Q Exactive MS instrument was used for the metabolomics analysis. Elution with a binary mobile phase was carried out at a flow rate of 0.25 mL/min. The column temperature was set at 40°C, and the injection volume was 2 μL. The mobile phases A and B were optimized for two different gradients. For the C18 column, phase A was 0.1% FA water, and phase B was 0.1% acetonitrile (0.1% FA). The gradient elution method was set as follows: 0–1 min, 2% B; 1–9 min, 2%–50% B; 9–12 min, 50%–98% B; 12–13.5 min, 98% B; 13.5–14 min, 98%–2% B; 14–20 min, 2% B.

The mass spectrometer used a dual jet stream electrospray ionization (ESI) source, the positive ionization (ESI^+^) source conditions were located as follows: spray voltages (3500 V), Sheath Gas (40 psi), capillary temperature (320°C), Auxiliary Gas (10 psi). Complete data acquisition was performed using the FULL MS/DD-MS2 (TOP5) mode. The full MS resolution was 70,000 FWHM, the AGC target was 5 × 10^6^, and the maximum injection time was 200 ms; MS/MS resolution was 17,500 FWHM, and the maximum injection time was 25 ms.

The raw spectra of UHPLC-Q Exactive MS were obtained by using Xcalibar (Thermo Fisher Scientific, Waltham, MA, United States). The original data were subjected to peak alignment, retention time correction, and peak area extraction using the Compound Discoverer 3.2 program. Information on ion fragmentation of unknowns was obtained. Qualitative metabolites were identified by retrieving the MZcloud and Chemspider databases and validated using HMDB databases (https://hmdb.ca/metabolites).

### 2.7 Fecal 16S rDNA amplicon sequencing and analysis

Microbial DNA was extracted from snap-frozen faecal samples of mice and DNA quality was determined by using agarose gel electrophoresis. The V3-V4 regions of the bacteria 16S ribosomal RNA gene were amplified by PCR using the flowing primers: CCTAYGGGRBGCASCAG and GGACTACNNGGGTATCTAAT. Then the PCR products were extracted from agarose gels and further purified and quantified. After the library was qualified, it was sequenced by Illumina MiSeq (GENEWIZ biological technology Co., Ltd., Suzhou, China). Amplicon Sequence Variants (ASVs) clustering and species classification analysis were then performed based on valid data. Species annotations were made for the representative sequences of each ASV, and the corresponding species information and species-based abundance distribution were obtained.

### 2.8 Fecal short-chain fatty acids LC-MS analysis

The faecal samples were resuspended with liquid nitrogen and then homogenized with methanol (80%) and centrifuged at 12,000 rpm for 10 min to remove the protein (Sun et al., 2023). The supernatant was added to derivatization reagent (150 μL) and derivatized at 40°C for 40 min. The sample was diluted by methanol (80%). Then supernatant (95 μL) was homogenized with 5 μL mixed internal standard solution.

An ultra-high performance liquid chromatography coupled to tandem mass spectrometry (UHPLC-MS/MS) system (Vanquish™ Flex UHPLC-TSQ Altis™, Thermo Scientific Corp., Germany) was used to quantitate SCFA in Novogene Co., Ltd. (Beijing, China). Separation was performed on a Waters ACQUITY UPLC BEH C18 column (2.1 × 100 mm, 1.7 μm) which was maintained at 40°C. The mobile phase, consisting of 10 mM ammonium acetate in water (solvent A) and acetonitrile: isopropanol (1:1) (solvent B), was delivered at a flow rate of 0.30 mL/min. The solvent gradient was set as follows: initial 25% B, 2.5 min; 25%–30% B, 3 min; 30%–35% B, 3.5 min; 35%–38% B, 4 min; 38%–40% B, 4.5 min; 40%–45% B, 5 min; 45%–50% B, 5.5 min; 50%–55% B, 6.5 min; 55%–58% B, 7 min; 58%–70% B, 7.5 min; 70%–100% B, 7.8 min; 100%–25% B, 10.1 min; 25% B, 12 min.

The mass spectrometer was operated in negative multiple reaction mode (MRM) mode. Parameters were as follows: IonSpray Voltage (−4500 V), Sheath Gas (35 psi), Ion Source Temp (550°C), Auxiliary Gas (50 psi), Collision Gas (55 psi).

### 2.9 Statistical analyses

Statistical analysis were performed by using IBM SPSS Statistics for Windows 26.0 and GraphPad Prism 9. The results are expressed in $\bar{X}$ ±SE. Differences were analyzed by Analysis of variance. *p* < 0.05 was considered statistically significant. SIMCA14.1 software (UMetrics AB, Umea, Sweden) was used to perform multidimensional statistical analysis, including unsupervised principal component analysis (PCA) and orthogonal partial least squares discriminant analysis (OPLS-DA). Data preprocessing: abnormal data and missing data were screened and processed, and then variables were normalized. To examine the quality of the models and to prevent overfitting of the models, cross-validation was performed on models, the KEGG pathway database was used to perform a channel enrichment analysis of the metabolites (https://www.metaboanalyst.ca/).

## 3 Results

### 3.1 The effects of semaglutide on weight, blood lipid and fasting blood glucose levels in obese mice and the rebound effect of semaglutide

Experimental results demonstrated that compared to the HFD group, the semaglutide intervention group showed a dose-dependent variation in weight gain trends (Figures 1B,C). The MSHF group significantly reduces the proportions of two types of white adipose tissues in obese mice (Figure 1D) and fasting blood glucose levels (Figure 1E) (*p <* 0.05). In comparison to the HFD group, the HSMF group significantly lowered the levels of TG and TC in obese mouse serum (*p* < 0.05); all three doses of semaglutide reduce LDL-C content, with the medium dose showing the most significant effect. There was no significant difference in the improvement of HDL-C levels across all groups (Figure 1F). After semaglutide intervention, mice showed reduced food intake, indicating that semaglutide could improve weight gain and reduce body fat induced by a high-fat diet by reducing energy intake. However, a comprehensive analysis of the changes in food intake throughout the entire experimental period did not show a clear dose dependency (Figure 1G).

Like many weight loss methods, it has been clinically observed that obese patients experience weight rebound after discontinuing semaglutide intervention (Wilding et al., 2022). Experimental results confirmed that 4 weeks after stopping semaglutide intervention, mice exhibit weight rebound and an increase in body fat percentage (Figures 1H–J). It is noteworthy that the medium dose MSHF group of semaglutide showed a significantly higher weight gain trend compared to the HFD group (*p* < 0.05). Analysis of the four blood lipid levels in mice showed that the LSHF group has significantly higher TG levels than the HFD group, while the MSHF group and HSHF group did not have a significant difference with HFD group. Overall, the semaglutide intervention groups showed a decreasing trend in TC, LDL-C, and HDL-C levels compared to the HFD group, but without significant differences (Figure 1K). Comparing fasting blood glucose levels in mice 4 weeks after stopping semaglutide injections, there was a decreasing trend in the semaglutide intervention groups compared to the HFD group, but without significant differences (Figure 1L). Observing the daily food intake of mice in each group, it can be found that after stopping semaglutide intervention, food intake increaseed in all groups and showed a dose-dependent relationship (Figure 1M).

Semaglutide could dose-dependently improve abnormal weight gain, body fat, blood lipid levels, and fasting blood glucose levels in obese mice. Additionally, after short-term semaglutide intervention discontinued, weight rebound, and a rise in body fat and blood glucose levels are observed. Overall, the HSHF group showed the most significant effects, and the dose of 100 μg/kg·bw of semaglutide was used in the subsequent experiments.

### 3.2 Semaglutide and fecal microbiota transplantation improved abnormal weight gain, body fat, blood lipid levels, fasting blood glucose, and insulin levels in obese mice, improving insulin resistance and impaired insulin sensitivity

Experimental results showed that the weight gain trend of the SHF group is close to that of the ND group and significantly different from the HFD group (*p* < 0.0001), while the MT group and FMT group showed a slowing of the weight gain trend (Figure 2B). Monitoring the daily food intake of mice revealed a significant decrease in food intake after semaglutide intervention (100 μg/kg·bw), with the FMT group showing a more significant decrease in daily food intake compared to the HFD group (Figure 2C) (*p* < 0.05). Semaglutide significantly improved the body weight gain rate and decreased body fat ratio in obese mice, with a modest improvement observed after fecal microbiota transplantation but not significant (Figures 2D,E). Compared to the HFD group, the SHF group significantly decreased TG, TC, and LDL-C levels, while increasing HDL-C levels (*p* < 0.05), indicating that semaglutide can improve blood lipid levels by reducing harmful factors and increasing beneficial factors. The MT group significantly decreased LDL-C levels, and the FMT group significantly decreased TG and LDL-C levels (*p* < 0.05), suggesting that bacterial transplantation not only reduces some cholesterol levels in the serum but also decreases triglyceride levels. However, the supernatant transplantation only showed some improvement in cholesterol levels (Figure 2F). Previous studies have shown that an increased HDL-C/LDL-C ratio is a negative risk factor for atherosclerosis (Wouters et al., 2005; Fruchart et al., 2004). Calculating the HDL-C/LDL-C ratio in mice from each group, the results indicated that the cardiovascular adverse effects of a high-fat diet can be significantly improved through semaglutide intervention (*p* < 0.05), and fecal microbiota transplantation also contributes to risk reduction, with the FMT group showing more significant improvement (Table 2).

**TABLE 2:** HDL-C/LDL-C ratio in mice.

Group	LDL-C	HDL-C	HDL-C/LDL-C
ND	1.12 ± 0.03^c^	2.68 ± 0.15^c^	2.38 ± 0.09^a^
HFD	3.65 ± 0.24^a^	3.03 ± 0.10^bc^	0.84 ± 0.03^c^
SHF	2.81 ± 0.22^b^	3.60 ± 0.12^a^	1.31 ± 0.07^b^
MT	2.63 ± 0.10^b^	3.12 ± 0.08^b^	1.20 ± 0.08^bc^
FMT	2.68 ± 0.29^b^	3.30 ± 0.13^ab^	1.35 ± 0.23^b^

Note: Significant differences are indicated by different letters (*p* < 0.05).

After 10 weeks of semaglutide intervention, the fasting blood glucose levels of mice in the SHF group were significantly lower compared to the HFD group (*p* < 0.05), with no significant difference compared to the ND group. The fasting blood glucose levels of mice in the MT group were also significantly lower than those in the HFD group, but the FMT group did not show a significant impact on the blood glucose elevation caused by a high-fat diet (Figure 2G). Additionally, the insulin levels of mice significantly decreased after semaglutide intervention (*p* < 0.05), and fecal microbiota transplantation replicated this improvement (Figure 2H). Calculating the homeostatic model assessment for insulin resistance (HOMA-IR) and homeostatic model assessment for insulin sensitivity (HOMA-IS) indices of mice based on fasting blood glucose and fasting insulin levels, it was found that compared to the ND group, the HOMA-IR of mice in the HFD group significantly increased, indicating insulin resistance. However, after semaglutide and fecal microbiota transplantation interventions, HOMA-IR significantly decreased compared to the HFD group. The HOMA-IS of mice in the HFD group was significantly lower than that of the ND group, indicating impaired insulin sensitivity. But after semaglutide and fecal microbiota transplantation interventions, HOMA-IS significantly increased compared to the HFD group (Figures 2I,J) (*p* < 0.05).

In conclusion, semaglutide and fecal microbiota transplantation have improved the abnormal weight gain, body fat increase, blood lipid levels, fasting blood glucose levels, and insulin levels in obese mice in varying degrees, as well as improved the levels of insulin resistance and impaired insulin sensitivity.

### 3.3 Semaglutide and fecal microbiota transplantation could reduce lipid accumulation in white adipose tissue and liver, and improve the expression of lipid metabolism-related genes in obese mice

**FIGURE 3:**
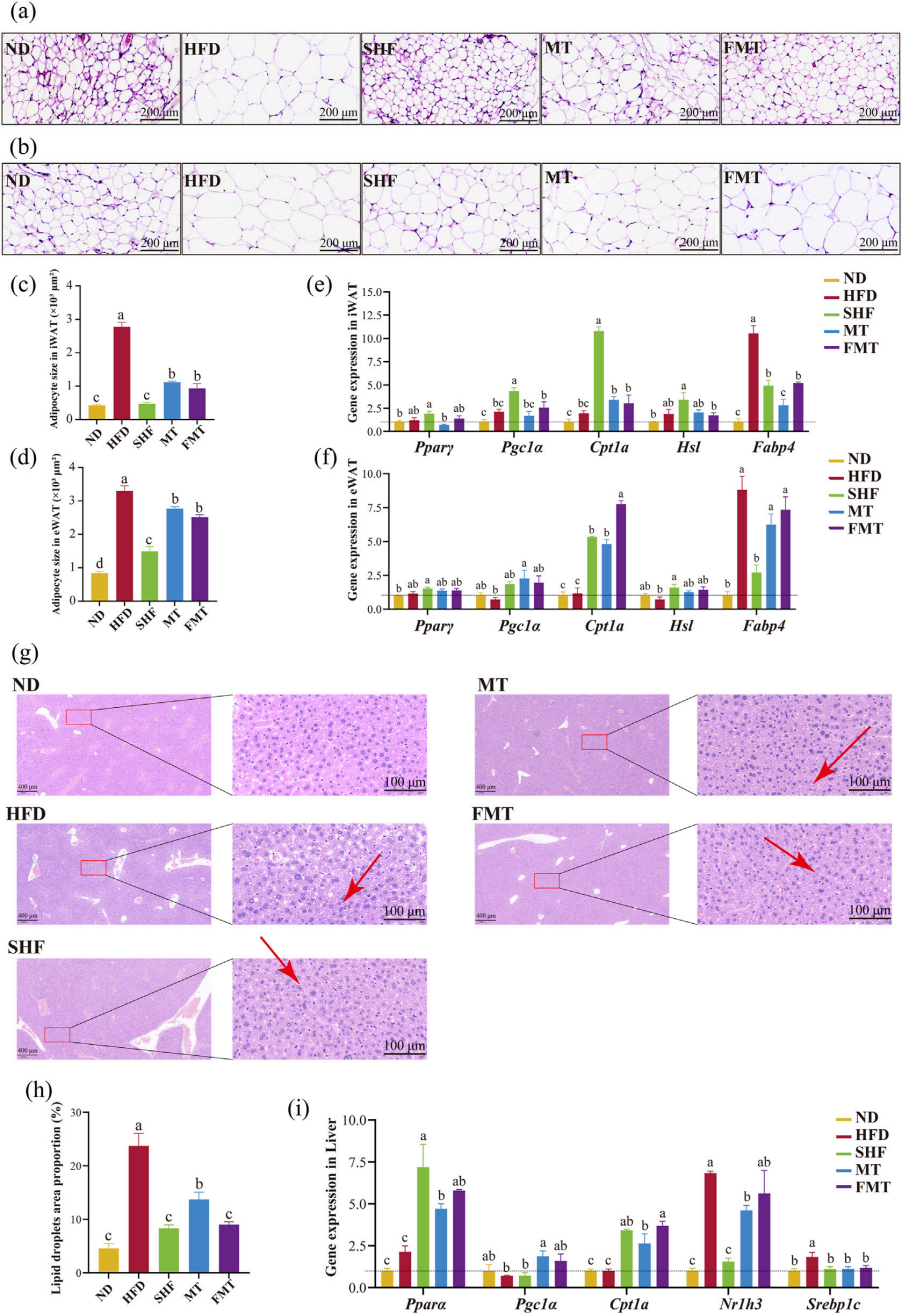
Effects of semaglutide and fecal microbiota transplantation on adipose tissue morphology and lipid metabolism in obese mice HE staining of iWAT (20 x) **(A)** and eWAT (20 x) **(B)**. Average area of adipocytes in iWAT **(C)** and in eWAT **(D)** (n = 5). Expression of genes involved in lipid metabolism-related genes in eWAT **(E)** and iWAT **(F)** (n = 4). HE staining results of liver (5 x and 40 x) **(G)**. Percentage of lipid droplet area in liver **(H)** (n = 5). Expression of genes involved in lipid metabolism related genes in liver **(I)** (n = 4). Note: Significant differences are indicated by different letters on the bar graph (*p* < 0.05), red arrows point to lipid droplet vacuoles.

The results of HE staining slices showed that compared to the ND group, the HFD group had significantly enlarged adipocyte areas with unclear boundaries between adipocytes. However, there was a visible improvement in the SHF, MT, and FMT groups (Figures 3A,B). The average adipocyte area of white adipose tissue in the inguinal and epididymal regions of mice in the HFD group was significantly higher than that of the ND group. In comparison to the HFD group, the SHF, MT, and FMT groups exhibited smaller and more uniform adipocyte morphology in both types of adipose tissue, with a significant decrease in the average adipocyte area (*p* < 0.05) (Figures 3C,D). Furthermore, the expression of lipid metabolism-related genes in the two types of white adipose tissue (iWAT and eWAT) was further evaluated. Compared to the ND group, the HFD group showed no significant difference in the expression of *Pparγ*, *Cpt1a*, *Pgc1α*, and *Hsl* in both types of adipose tissue, while the expression of *Fabp4* significantly increased (*p* < 0.05). After semaglutide intervention, the expression levels of *Pparγ*, *Cpt1a*, *Pgc1α*, and *Hsl* in obese mice significantly increased (*p* < 0.05), while the expression level of *Fabp4* significantly decreased (*p* < 0.05). Following fecal microbiota transplantation intervention, both the MT and FMT groups showed a significant decrease in the expression level of *Fabp4* in iWAT and a significant increase in the expression level of *Cpt1a* (Figure 3E). Additionally, the MT group showed a significant increase in the expression level of *Pgc1α* in eWAT, while the FMT group only showed a trend of increase without significant difference (Figure 3F).

The results of HE staining of mouse liver showed that the HFD group had a large number of unevenly sized lipid droplets in the liver; the SHF, MT, and FMT groups also exhibited some unevenly sized lipid droplets in the liver. HE staining revealed that in the high-fat diet group, there was a large number of uniformly sized small lipid droplets infiltrating the cytoplasm around the central vein, and a small amount of large vacuoles filled with lipid due to macrovesicular steatosis around the portal area. However, after intervention with semaglutide and fecal microbiota transplantation, both microvesicular and macrovesicular steatosis were reduced (Figure 3G). The area percentage of lipid droplets in the liver of the HFD group was significantly higher than that of the ND group. After intervention with semaglutide and fecal microbiota transplantation, the area percentage of lipid droplets in the liver of mice significantly decreased (Figure 3H) (*p* < 0.05). The expression of lipid metabolism-related genes in the liver showed that compared to the ND group, the HFD group had no significant difference in the expression of *Pparα*, *Cpt1a*, and *Pgc1α*, while the expression levels of *Srebp1c* and *Nr1h3* significantly increased (*p* < 0.05); the SHF group showed a significant increase in the expression levels of *Pparα* and *Cpt1a* (*p* < 0.05), a significant decrease in the expression levels of *Srebp1c* and *Nr1h3* (*p* < 0.05), and no significant difference in the expression of *Pgc1α*; additionally, the MT group, which received the supernatant transplantation, significantly increased the expression of *Pparα, Cpt1a*, and *Pgc1α*, and decreased the expression of *Srebp1c* and *Nr1h3* (*p* < 0.05); the FMT group, which received the bacterial transplantation, significantly increased the expression of *Pparα* and *Cpt1a*, and decreased the expression of *Srebp1c* (*p* < 0.05) (Figure 3I).

The previous experimental results showed that semaglutide has the effect of reducing daily food intake. Therefore, the expression of appetite-regulating genes in the hypothalamus was further evaluated. In hypothalamic, there was no significant difference in the expression of *Leptin*, *Agrp*, and *Pomc* between the HFD group and ND group, while the expression level of *Npy* significantly increased (*p* < 0.05) in the HFD group. After semaglutide intervention, the SHF group showed a significant decrease in the expression levels of *Agrp* and *Npy* (*p* < 0.05), with no significant difference in the decreasing trend of *Leptin* expression and increasing trend of *Pomc* expression. The MT group, which received the supernatant transplantation, significantly increased the expression of *Leptin* and *Pomc* (*p* < 0.05). The FMT group, which received the bacterial transplantation, significantly decreased the expression of *Leptin* and *Npy* (*p* < 0.05) (Supplementary Figure S2A).

In summary, semaglutide and fecal microbiota transplantation could reduce lipid accumulation in white adipose tissue and liver by enhancing the expression of lipid oxidation-related factors and inhibiting the expression of lipid synthesis-related factors, thus improving the metabolic abnormalities of obese mice.

### 3.4 The benefits of semaglutide and fecal microbiota transplantation in improving metabolic abnormalities induced by a high-fat diet were confirmed by using non-targeted metabolomics techniques

**FIGURE 4:**
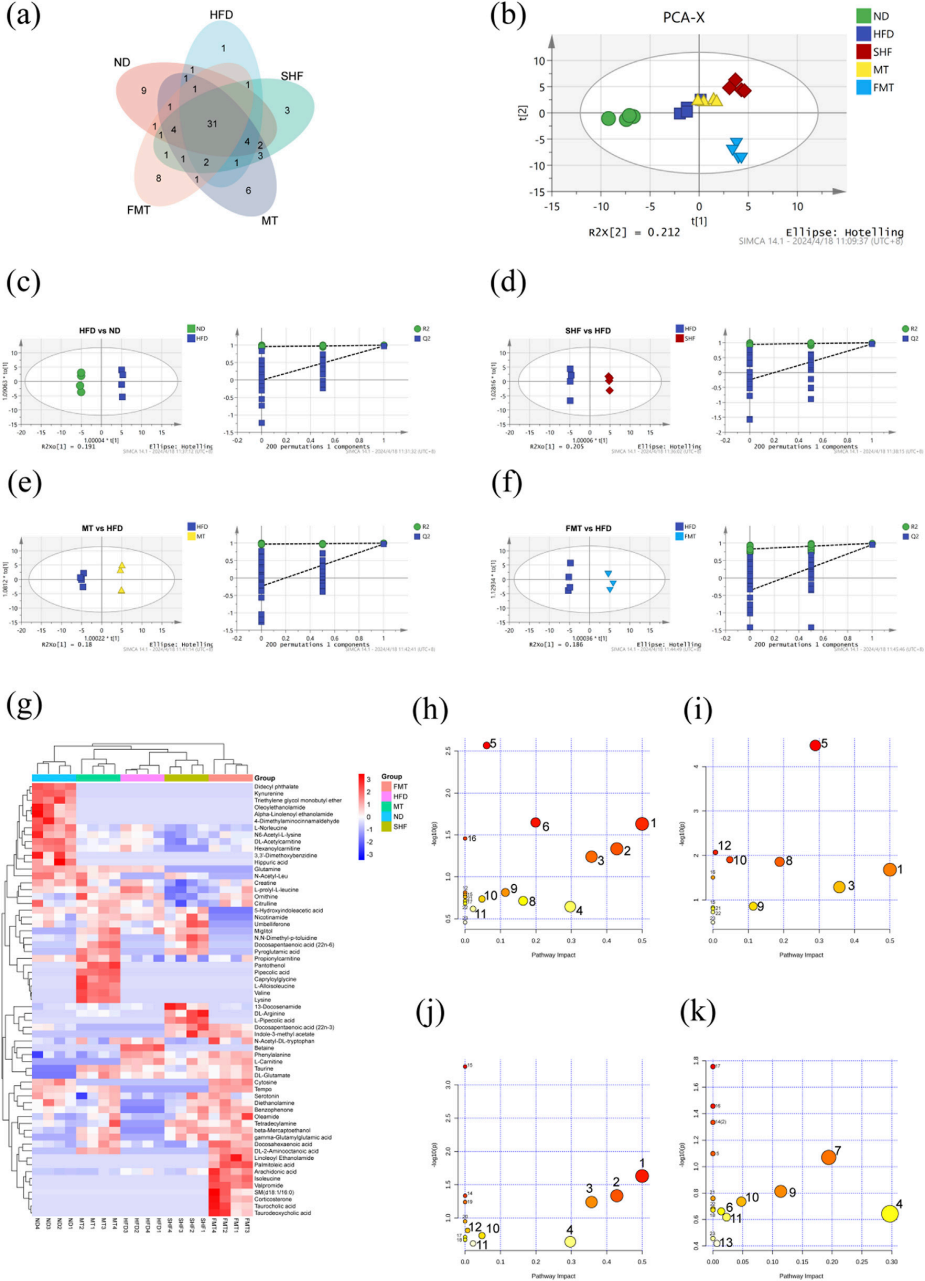
Effect of semaglutide and fecal microbiota transplantation on serum metabolism in obese mice (n = 4) Venn diagram **(A)**. PCA plot **(B)**. OPLSDA and replacement test for HFD vs. ND **(C)**, SHF vs. HFD **(D)**, MT vs HFD **(E)**, FMT vs. HFD **(F)**. Heat map of differential metabolite clustering **(G)**. Bubble plots of pathway enrichment for HFD vs. ND **(H)**, SHF vs. HFD **(I)**, MT vs. HFD **(J)**, FMT vs. HFD **(K)**.

Total ion chromatograms (TIC) of different groups of mouse serum metabolites were obtained through UHPLC-QExactive MS/MS non-targeted metabolomics techniques, showed significant differences in metabolite levels among the groups (Supplementary Figure S2B). By filtering and selecting the metabolic characteristics of each sample, a total of 84 serum metabolites were identified (Supplementary Table S1). A venn diagram illustrated the commonalities and differences in serum metabolites among the groups of mice (Figure 4A). In the PCA model, it was able to completely classify and identify the serum of mice from five different intervention groups, indicating a significant impact of diet, semaglutide, and fecal microbiota transplantation on the serum metabolome of mice (Figure 4B). Therefore, pairwise comparisons of serum metabolite spectra among the five groups of mice were conducted, OPLS-DA models were constructed, and the reliability of the models was tested 200 times. Analyzing the OPLS-DA models and permutation tests of ND vs. HFD, HFD vs. SHF, HFD vs. MT, and HFD vs. FMT revealed clear distinctions among the groups with good permutation test results (Figures 4C–F). By selecting potential feature metabolites among the samples based on VIP>1 and *p* < 0.05, a heatmap of differential metabolites among the groups was generated, showing that the ND group mainly focused on metabolites such as α-linolenoyl ethanolamide, uric acid, and N6-acetyl-L-lysine; the HFD group mainly concentrated on metabolites like betaine, phenylalanine, and taurine; the SHF group mainly centered around metabolites like DL-arginine, L-pipecolic acid, and indole-3-methyl acetate; the MT group mainly showcased metabolites such as L-isoleucine, valine, and lysine; and the FMT group mainly highlighted metabolites like isoleucine, corticosterone, and taurodeoxycholic acid (Figure 4G). By conducting KEGG metabolic pathway enrichment analysis on the differential metabolites of each group, it can be observed that a high-fat diet affects the biosynthesis of phenylalanine, tyrosine, and tryptophan, taurine and hypotaurine metabolism, phenylalanine metabolism, and arachidonic acid metabolism in mice. Semaglutide intervention affects the biosynthesis of phenylalanine, tyrosine, and tryptophan, phenylalanine metabolism, arginine biosynthesis, and arginine and proline metabolism. Transplantation of supernatant affects the biosynthesis of phenylalanine, tyrosine, and tryptophan, taurine and hypotaurine metabolism, phenylalanine metabolism, and arachidonic acid metabolism. Transplantation of bacteria affects arachidonic acid metabolism, niacin and nicotinamide metabolism, alanine, aspartate and glutamate metabolism, and glycine, serine, and threonine metabolism (Figures 4H–K; Table 3).

**TABLE 3:** Metabolic pathways enrichment results.

No.	Pathway Name	HFD vs. ND	SHF vs. HFD	MT vs. HFD	FMT vs. HFD
1	Phenylalanine, tyrosine and tryptophan biosynthesis	√	√	√	
2	Taurine and hypotaurine metabolism	√		√	√
3	Phenylalanine metabolism	√	√	√	
4	Arachidonic acid metabolism	√		√	√
5	Arginine biosynthesis	√	√		√
6	Tryptophan metabolism	√			√
7	Nicotinate and nicotinamide metabolism				√
8	Arginine and proline metabolism	√	√		
9	Alanine, aspartate and glutamate metabolism	√	√		√
10	Glycine, serine and threonine metabolism	√	√	√	√
11	Primary bile acid biosynthesis	√		√	√
12	Glutathione metabolism	√	√	√	
13	Steroid hormone biosynthesis				√
14	Valine, leucine and isoleucine biosynthesis			√	√
15	Lysine degradation	√	√	√	
16	Nitrogen metabolism	√	√		√
17	Biosynthesis of unsaturated fatty acids	√		√	√
18	Valine, leucine and isoleucine degradation			√	√
19	Biotin metabolism			√	
20	Pantothenate and CoA biosynthesis			√	
21	Glyoxylate and dicarboxylate metabolism	√	√		√
22	Pyrimidine metabolism	√	√		√
23	Purine metabolism	√	√		√

### 3.5 Semaglutide intervention and fecal microbiota transplantation improved the dysbiosis of gut microbiota in obese mice, increasing the relative abundance of beneficial bacteria, and affecting the levels of short-chain fatty acids

**FIGURE 5:**
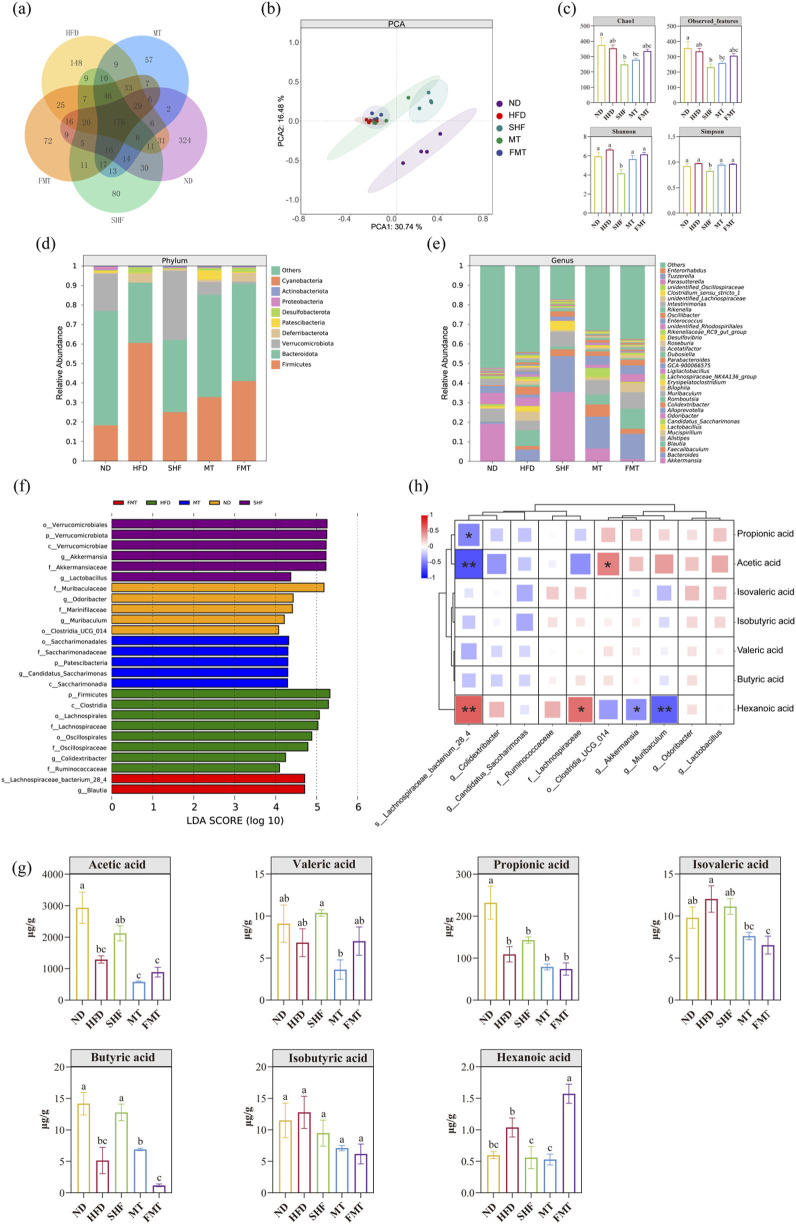
Effects of semaglutide and fecal microbiota transplantation on gut microbiota in obese mice (n = 4) Vann diagram. PCA plot. α-diversity. Histogram of relative abundance of species at the phylum level and genus level. LDA analysis, Vann diagram **(A)**. PCA plot **(B)**. α-diversity **(C)**. Histogram of relative abundance of species at the phylum level **(D)** and genus level **(E)**. LDA analysis **(F)**. Short-chain fatty acid level **(G)**. Correlation analysis of differential species and short-chain fatty acid levels **(H)** Note: Significant differences are indicated by different letters on the bar graph,*p < 0.05,**p < 0.01.

The species accumulation curve and dilution curve indicated that the sample size and sequencing depth were sufficient (Supplementary Figure S3A). The venn diagram showed the number of Amplicon Sequence Variants (ASVs) of gut microbiota in the five groups of mice (Figure 5A). PCA analysis results revealed that under different dietary backgrounds, the ND group was clearly separated from the other groups with no intersection. Under a high-fat diet background, the SHF group was somewhat separated from the HFD group, suggesting that semaglutide intervention could improve the composition of gut microbiota in obese mice (Figure 5B). The α-diversity index showed that the diversity and richness of microbiota in mice significantly decreased after semaglutide intervention (*p* < 0.05). The reason may be the decrease in daily food intake leading to changes in gut microbiota diversity. Additionally, the gut microbiota of the MT group and FMT group were similarly disrupted after antibiotic treatment, with the microbial body transplantation recovering the gut microbiota faster than the supernatant transplantation (Figure 5C). Analysis at the phylum and genus levels of relative species abundance histograms revealed that the abundance of the top ten bacterial genera at the phylum level in each group mainly focused on Firmicutes, Bacteroidota, Verrucomicrobiota, and Deferribacterota, etc. (Figure 5D). The abundance of the top thirty-five bacterial genera in each group mainly concentrated on *Akkermansia*, *Bacteroides*, *Faecalibaculum*, *Blautia*, and *Mucispirillum*, etc. (Figure 5E). Using LEfSe analysis to find statistically different biomarkers between the five groups, the bar chart of LDA score distribution showed species with LDA scores greater than 4. Selecting the minimum classified units in each group, the characteristic bacteria of the ND group were *Muribaculum spp.*, *Odoribacter spp.*, and *Clostridia-UCG-014*; the characteristic bacteria of the HFD group were Lachnospiraceae, Ruminococcaceae, and *Colidextribacter spp*.; the characteristic bacteria of the SHF group were *Akkermansia spp.* and *Lactobacillu spp.*; the characteristic bacteria of the MT group were *g_Candidatus_Saccharimonas*; and the characteristic bacteria of the FMT group were *Lachnospiraceae bacterium*_28_4 (Figure 5F). Analysis of short-chain fatty acid content in mouse feces revealed that the levels of acetic acid, propionic acid, and butyric acid in HFD mice were lower than in the normal group (*p* < 0.05), and this unfavorable trend improved after semaglutide intervention; after fecal microbiota transplantation intervention, the levels of short-chain fatty acids in obese mice also changed (Figure 5G). Spearman correlation analysis between the characteristic bacteria of each group and short-chain fatty acids showed that, except for valeric acid, the other short-chain fatty acids exhibited similar correlations. Dominant bacteria in normal mice and mice after semaglutide intervention were significantly positively correlated with the content of short-chain fatty acids, while dominant bacteria in high-fat diet mice were significantly negatively correlated with the content of short-chain fatty acids (Figure 5H). The study also found correlations between dominant bacteria in each group and certain predicted metabolic functions, such as oxidative phosphorylation, metabolism of glycine, serine, and threonine, arginine and proline metabolism, ketone body metabolism, glycolysis/gluconeogenesis, pentose phosphate pathway, fructose and mannose metabolism, starch and sucrose metabolism, and methane metabolism, etc. (Supplementary Figure S3B).

In summary, semaglutide and fecal microbiota transplantation improved the dysbiosis of gut microbiota in obese mice by increasing the relative abundance of beneficial bacteria, and influencing the levels of short-chain fatty acids.

## 4 Discussion

The improvement of semaglutide on obese individuals is significant, and the main mechanism of this type of GLP-1RA is to stimulate GLP-1R, triggering activation of several metabolic pathways involved in insulin secretion, lipid metabolism, energy expenditure, cell survival, anti-apoptotic signaling, and oxidative stress prevention in tissues such as the pancreas, central nervous system, heart, muscles, kidneys, and intestines (Rowlands et al., 2018). Prior studies on the effects of semaglutide on gut microbiota in high-fat diet-induced obese mice and whether fecal transplantation can transmit the weight-loss effects of semaglutide are not yet clear.

We found that intraperitoneal injection of 100 μg/kg·bw of semaglutide every other day significantly improved the abnormal weight gain induced by a high-fat diet, reduced the proportion of white adipose tissue in the groin and epididymis; serum levels of TG, TC, and LDL-C in mice were significantly reduced, while HDL-C levels were significantly increased, improving the adverse cardiovascular effects of a high-fat diet; fasting blood glucose levels and insulin resistance were also significantly improved. Insulin resistance is the inadequate response of target tissues to the physiological effects of circulating insulin, such as decreased glucose uptake in skeletal muscles under insulin stimulation, inhibition of hepatic gluconeogenesis by insulin, and relief of the inhibitory effect of insulin on lipid breakdown in adipose tissue (Schenk et al., 2008). Initially, when insulin resistance occurs, pancreatic bcells compensate by secreting more insulin to maintain normal glucose metabolism, but as the disease progresses, pancreatic b cell function deteriorates, insulin secretion significantly decreases, blood sugar cannot return to normal baseline levels, leading to metabolic disorders, including glucose and lipid abnormalities, and metabolic diseases such as T2DM (Warram et al., 1990; Lillioja et al., 1988). Semaglutide replaces GLP-1 and stimulates insulin secretion through the corresponding receptor, increasing insulin secretion, regulating blood glucose levels, and promoting appropriate weight loss.

The main cause of the prevalence of obesity is the long-term imbalance between calorie intake and expenditure. HE staining of slices from iWAT, eWAT and liver in mice revealed that semaglutide significantly improved the enlargement of fat cells caused by a high-fat diet and reduced lipid accumulation in liver. However, using hematoxylin and eosin (HE) staining in evaluating liver tissues presents certain limitations for accurately assessing steatosis. Additionally, the determination of serum TG, TC, LDL-C, and HDL-C levels alone is insufficient for the diagnosis of fatty liver disease. To comprehensively evaluate the therapeutic effects of semaglutide on hepatic steatosis, future studies should incorporate complementary methods such as Oil Red O staining (Zhu et al., 2019), biochemical hepatic TG assays (Rong et al., 2024), or non-invasive imaging techniques such as magnetic resonance imaging with proton density fat fraction (MRI-PDFF) (Park et al., 2022).

GLP-1RA is associated with reduced food intake and weight loss, and its molecular mechanism involves the stimulation of various signaling pathways by GLP-1R. Activation of the PPAR pathway negatively affects fat formation by downregulating the expression of genes associated with new fat formation. These downregulated genes include *Fabp4* (Furuhashi and Hotamisligil, 2008), S*ebp1c* (Ferre et al., 2021; Musso et al., 2009; Hagiwara et al., 2012), *Nr1h3* (Cariello et al., 2021; Viscarra and Sul, 2020), which are involved in the synthesis of fatty acids and triglycerides. GLP-1RA also involves lipid solubilization processes, downstream proteins related to fatty acid oxidation such as CPT-1A (Zheng et al., 2011), PGC1α (Jamwal et al., 2021), and key enzymes involved in triglyceride breakdown and lipolysis, such as HSL, are also regulated by PPARs (Tian et al., 2013), leading to reduced fat accumulation and increased energy expenditure.

This study found that semaglutide significantly upregulated the levels of *Pparγ* and downstream genes *Cpt-1a*, *Pgc1α*, and *Hsl* in adipose tissue, and *Ppar*α, *Cpt-1a*, *Pgc1α* in the liver; significantly downregulated the levels of *Fabp4* in adipose tissue and *Srebp1c*, *Nr1h3* in the liver, promoting triglyceride hydrolysis and fatty acid oxidation in adipose tissue and liver of obese mice, inhibiting the fatty acid synthesis pathway to improve lipid metabolism. The study also found that after semaglutide intervention, the food intake of obese mice decreased significantly, achieved by regulating appetite-related factors in the hypothalamus, indicating that semaglutide could penetrate the blood-brain barrier to reach the hypothalamus, brainstem, and arcuate nucleus through peripheral administration (Martins et al., 2022). Approximately 10% of POMC^+^ neurons in the arcuate nucleus co-express GLP-1R and leptin receptors, and chemogenetic activation of POMC^Glp1r+^ neurons leads to reduced food intake in male mice (Biglari et al., 2021).

In addition to regulating appetite, the semaglutide also affects insulin secretion, blood glucose homeostasis, energy expenditure, and lipid metabolism processes, as well as serum metabolism. To understand the impact of semaglutide on serum metabolism, we conducted a non-targeted metabolomics analysis. Semaglutide intervention affects the biosynthesis of phenylalanine, tyrosine, and tryptophan, phenylalanine metabolism, arginine biosynthesis, as well as arginine and proline metabolism in obese mice. The amino acid sequence of semaglutide itself and the fatty acid side chain act as metabolic substrates after intestinal absorption, affecting the body’s metabolism. Research has shown that even non-GLP-1RA agents have appetite-suppressing effects and can improve obesity (Li et al., 2023). Changes in amino acid metabolism reflect the metabolic health status of obese populations, especially changes in the blood concentrations of specific essential amino acids and their derivatives (particularly branched-chain amino acids, sulfur amino acids, tyrosine, and phenylalanine) (Bagheri et al., 2018). In metabolomics studies, differences in the concentration of phenylalanine and its first breakdown product tyrosine are factors that distinguish obese from non-obese human participants (Newgard et al., 2009) or compare insulin-sensitive and insulin-resistant individuals (Tai et al., 2010), and are associated with the persistence of childhood obesity (Wu et al., 2024). Research has shown that aromatic amino acids (including tryptophan, phenylalanine, and tyrosine) affect bile acid synthesis and lipid metabolism (Chen et al., 2024). Additionally, amino acid metabolism is closely related to gut microbiota. For example, tryptophan, an amino acid closely related to obesity (Natividad et al., 2018), can be broken down by gut bacteria into indole and its derivatives, participating in the pathways of urine amino acid metabolism (Agus et al., 2018). It can be seen that as a GLP-1RA, semaglutide can reduce the risk of obesity by regulating serum organic acid and lipid metabolism, thereby balancing the body in the context of obesity in mice.

The gut microbiota is increasingly recognized as a key driving factor in obesity. The composition of the gut microbiota depends on many factors, such as the gastrointestinal region, host genetics, nutrition and health status, dietary habits, and the use of antibiotics. Research has shown that the activation of GLP-1R can lead to a reduction in chylomicron (CM) secretion in postprandial intestinal cells (Hsieh et al., 2010), and the gain and loss of GLP-1R signal transduction can regulate mucosal expansion in the small intestine and colon, affecting intestinal integrity and systemic inflammation (Koehler et al., 2015). Studies have found that liraglutide can improve intestinal epithelial integrity, reduce increased intestinal permeability (Nozu et al., 2018), and it is speculated that semaglutide has similar functions and affects gut microbiota balance. This study found that semaglutide improves the dysbiosis of gut microbiota in obese mice, increasing the relative abundance of beneficial bacteria. Following semaglutide intervention, the relative abundances of *Akkermansia* and *Lactobacillus* significantly increased, making them dominant beneficial bacteria in improving gut microbiota with semaglutide intervention. Semaglutide intervention significantly upregulated the representative species of the phylum Verrucomicrobia, *Akkermansia muciniphila*. On one hand, it can degrade intestinal mucin glycoproteins by enzymes (such as glycoside hydrolases, proteases, sulfatases, and sialidases) to produce SCFAs, acetates, propionates, and 1,2-propanediol, as well as succinates and sulfates, promoting mucin turnover and thickening, thereby enhancing intestinal barrier function and reducing the permeability of gut microbiota products; another barrier-enhancing mechanism is where *Akkermansia* induces Paneth cells to produce antimicrobial peptides, and SCFAs from intestinal mucin glycoproteins are absorbed in the colon as an energy source for colonic cells, inducing regulatory T cells and exerting anti-inflammatory effects (Everard et al., 2013; Reunanen et al., 2015; Belzer and de Vos, 2012; Ottman et al., 2017; Brodmann et al., 2017). Studies have found that obesity is associated with microbiota-induced chronic low-grade inflammation (Hotamisligil, 2017).

Obesity and obesity-related diseases are a major public health issue worldwide. In addition to traditional weight loss drugs, probiotic therapy showed great potential. *Akkermansia muciniphila* and *Lactobacillus* are common probiotics that can produce SCFAs and are associated with anti-inflammatory effects (Wang et al., 2023), maintaining intestinal barrier function (Liang et al., 2023) and lipid metabolism (Ma et al., 2022). They can improve epithelial barrier function, regulate immune responses, and are commonly used to prevent obesity and treat intestinal dysbiosis (Li et al., 2020; Song et al., 2021). Research has shown that a calorie-restricted diet pattern quickly leads to a microbiota dominated by *Lactobacillus* in mice (Pan et al., 2018). Furthermore, levels of SCFAs have changed after semaglutide intervention, and the types and amounts of SCFAs in the body are closely related to gut microbiota (Wang et al., 2020). Correlation analysis shows that dominant microbiota under semaglutide intervention is significantly associated with the production of SCFAs, and functional predictions suggest that gut microbiota is also related to amino acid metabolism and lipid metabolism. As the site of GLP-1 secretion, the stability of the gut microbiota itself affects the peripheral circulation of GLP-1, and their interaction forms a robust regulatory pathway. The gut microbiota, through interactions with host cell receptors and metabolites produced, activate or inhibit signaling pathways, regulating many different aspects of metabolic diseases.

However, analysis of the gut microbiota after semaglutide intervention showed a decrease in diversity and richness. Research has shown that calorie-restricted diet interventions in random human subjects, while improving metabolic health, lead to a reduction in bacterial numbers and a reshuffling of the gut microbiota due to strict calorie restrictions (von Schwartzenberg et al., 2021). Similar results have been observed in mice (Pan et al., 2018). Therefore, the significant reduction in daily food intake after semaglutide intervention may be one of the reasons for the damage to the diversity and richness of the gut microbiota.

The improvement effect of semaglutide on obese mice is partially driven by changes in serum metabolism and gut microbiota, as confirmed by fecal transplant experiments in this study. Transplanting the gut microbiota of mice in the semaglutide intervention group to recipient mice can reduce fasting blood glucose and insulin levels, improve insulin resistance, reduce lipid accumulation in white adipose tissue and liver, and regulate levels of lipid metabolism-related factors. However, although the mice undergoing fecal transplant showed a slower increase in body weight compared to the high-fat diet (HFD) group, there was no significant difference. Furthermore, the fecal transplant did not replicate the effect of semaglutide in improving body fat in terms of the apparent index. It is speculated that the effect of fecal transplant may have a lagging nature, and the improvement in serum biochemical indicators may not be reflected in the apparent body weight and body fat.

In recent years, the role of gut microbiota in health and disease has become a hot topic. Fecal microbiota transplantation is a targeted therapy that reconstructs the recipient’s gut microbiota by transplanting fecal microbiota from donors to the recipient’s gastrointestinal tract. Currently, research on animal experiments of fecal transplant focuses more on the intrinsic states of donor and recipient mice, additional drug interventions, and transplantation methods (Porcari et al., 2023). There is a consensus on the handling of transplant materials, but there is limited research on the efficacy of metabolites in fecal suspension supernatant transplantation. This study found that fecal microbiota transplantation can transfer the beneficial effects of semaglutide in improving obesity to recipient mice. However, the effects of transplanting supernatant and bacteria are different, with different manifestations in changes in serum metabolism and regulation of gut microbiota. Compared to the HFD group, the FMT group with bacteria transplantation identified 29 differential metabolites, while the MT group only identified 24 differential metabolites. The MT group mainly affected the metabolism pathway of phenylalanine, while the FMT group had more impact on arachidonic acid metabolism, niacin and niacinamide metabolism, and metabolism of alanine, aspartate, and glutamate.

In terms of gut microbiota, both transplanting supernatant and bacteria could lower the F/B ratio of the gut microbiota in obese mice and regulate the composition of bacterial genera. LEfSe analysis showed that one of the dominant genera in the MT group was *Candidatus_ Saccharimonas*, which has been associated with inflammation, lipid metabolism, and oxidative stress (Yang et al., 2023). It is a beneficial microbiota in fructooligosaccharide treatment for diabetic nephropathy (Luo et al., 2022). The dominance of *Ruminococcaceae_bacterium* GD6 in this genus has been reflected in related studies on ulcerative colitis (Xie et al., 2023). One of the dominant genera in the FMT group was the potential probiotic *Blautia*, which, when orally administered to mice, induces metabolic changes and anti-inflammatory effects, reducing obesity and diabetes caused by a high-fat diet, its beneficial effects are related to unique amino acid metabolism (Hosomi et al., 2022). Therefore, through fecal microbiota transplantation technology, it has been confirmed that changes in gut microbiota are more significant in improving obesity than intestinal metabolites, indicating that the beneficial effects of semaglutide on obese mice are more driven by changes in the gut microbiota.

Research has found that semaglutide improves high-fat diet-induced obesity in mice in a dose-dependent manner. When the intervention is stopped, mice food intake increases again, and their weight rebounds. The rebound in body weight is related to increased food intake, lipid metabolism (Zhong et al., 2022), the persistent presence of immune cell phenotypes in adipose tissue (van Baak and Mariman, 2023) and adipocyte stress (Van Baak and Mariman, 2019). The rebound phenomenon has always been a key focus in evaluating the effectiveness of weight loss methods. Research has shown that 18 weeks of liraglutide intervention can possibly be related to reducing proliferation and inflammation of hypothalamic microglia in resetting the body weight set point (BWSP) of diet-induced obese (DIO) rats (Liao et al., 2020). It is speculated that semaglutide reduces mice’s food intake to achieve a negative energy balance state, and the accompanying weight loss triggers the formation of compensatory physiological mechanisms. The body’s BWSP remains unchanged, and after normal food intake is resumed, the compensatory physiological mechanisms can remain activated, promoting weight rebound.

An important component of GLP-1RA treatment is lifestyle intervention, including exercise and low-calorie diet, which is crucial for weight maintenance. Therefore, long-term and persistent intervention with semaglutide is needed for the improvement of obesity. This study identified changes in key metabolites such as phenylalanine and amino acids, as well as key bacterial genera *Akkermansia* and *Lactobacillus* after semaglutide intervention, but verification experiments were not conducted. Therefore, further exploration is needed on how to improve the phenomenon of weight rebound after stopping semaglutide intervention, other pathways affected by semaglutide, and whether improvement in obesity can be achieved through direct intervention with key metabolites/key bacterial genera.

## Data Availability

The original contributions presented in the study are publicly available. This data can be found here: https://www.scidb.cn/en/anonymous/bWE2Qlpi.
