# Supplementary material for: Effects of semaglutide on metabolism and gut microbiota in high-fat diet-induced obese mice
Source: Front Pharmacol. 2025 Jun 2;16:1562896. doi: 10.3389/fphar.2025.1562896 (PMC12183439; doi:10.3389/fphar.2025.1562896)
Supplement: Supplementary file 1 [file DataSheet1.docx]

**Supplementary materials**

Supplementary (2.4 Adipose Tissue and Liver Morphology)

Image analysis-based quantification of steatosis and adipocytes：The histological effects of different interventions on mice were evaluated by measuring the average size of adipocytes in adipose tissue and the average area of lipid droplets in liver tissue. ImageJ software (NIH, Bethesda, MD, United States) was used. Five fields of view (20x) of adipose tissue sections stained and five fields of view (40x) of liver tissue sections stained were selected for measurement. The average size of adipocytes was calculated as the cell area in each field divided by the cell count, and the area of lipid droplets in the liver was calculated as the droplet area in each field divided by the field area and multiplied by 100%. The images were converted to 8-bit and the brightness was adjusted, with the threshold set at 200~255 to ensure the red area covered the fat/lipid droplets and the boundaries were clear. For the adipocyte part, a suitable lower limit of size (100) was set to eliminate the influence of tiny particles. For the liver lipid droplets, the circularity was set at 0.70~1.00 to exclude the influence of sinusoids, blood vessels, and other non-lipid droplet parts. Clicking "Measure" would obtain the overall area, and clicking "Analyze Particles" would get the area of individual cells. The data were exported for subsequent data analysis. The ImageJ macros are coded as follows, along with representative example images (see Supplementary Figure 1).

| Macro Code(Adipose) | Macro Code(Liver) |
| --- | --- |
| open("xxx.tif");  run("8-bit");  setAutoThreshold("Default dark no-reset");  //run("Threshold...");  //setThreshold(200, 255);  setOption("BlackBackground", true);  run("Convert to Mask");  run("Analyze Particles...", "size=100-10000 display clear include summarize"); | open("xxx.tif");  run("8-bit");  setAutoThreshold("Default dark no-reset");  //run("Threshold...");  //setThreshold(200, 255);  setOption("BlackBackground", true);  run("Convert to Mask");  run("Analyze Particles...", "size=0-100 circularity=0.7-1.00 show=Masks display exclude clear include summarize"); |


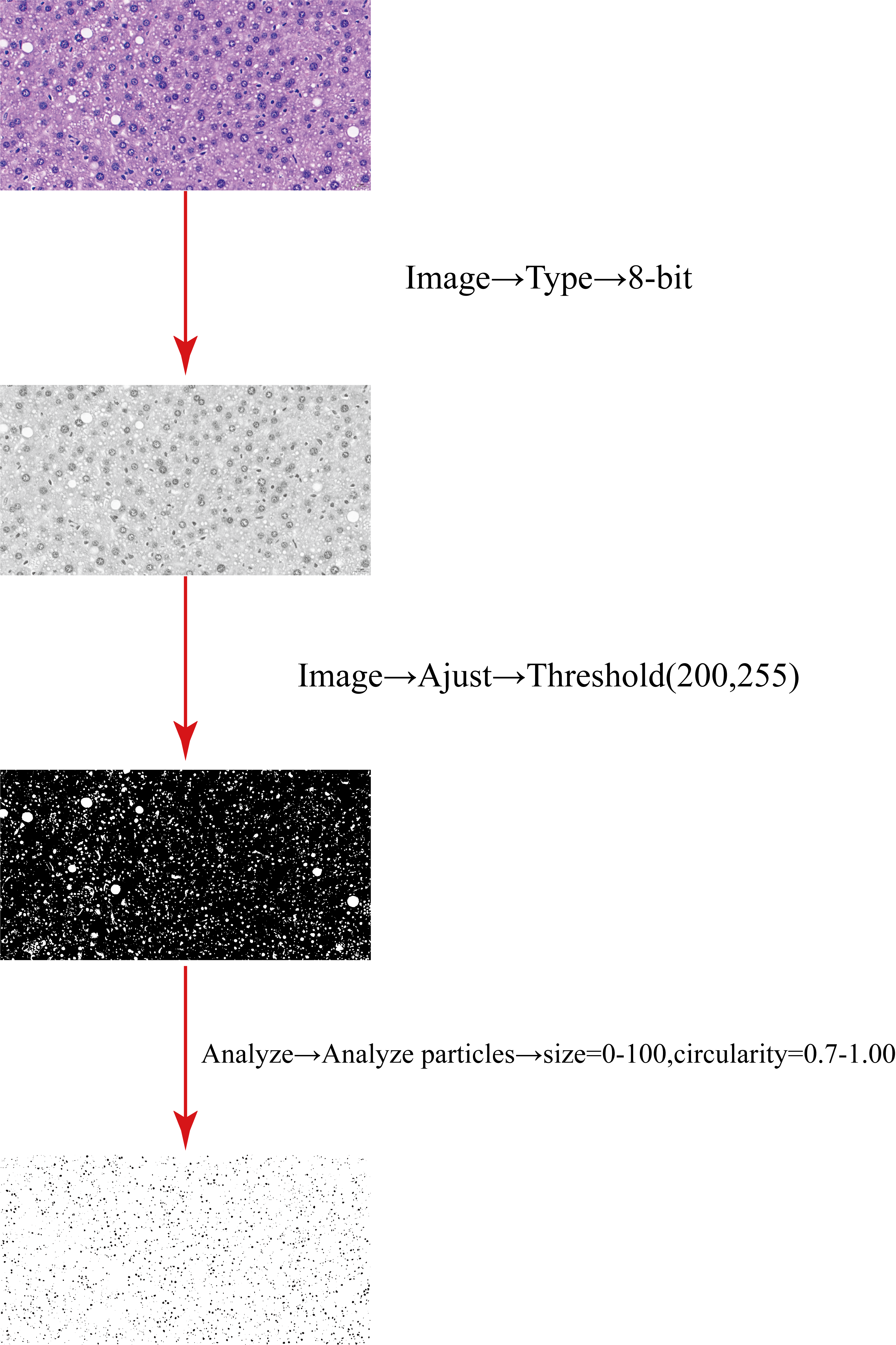
 **Supplementary Figure 1**

Example image of representative ImageJ.


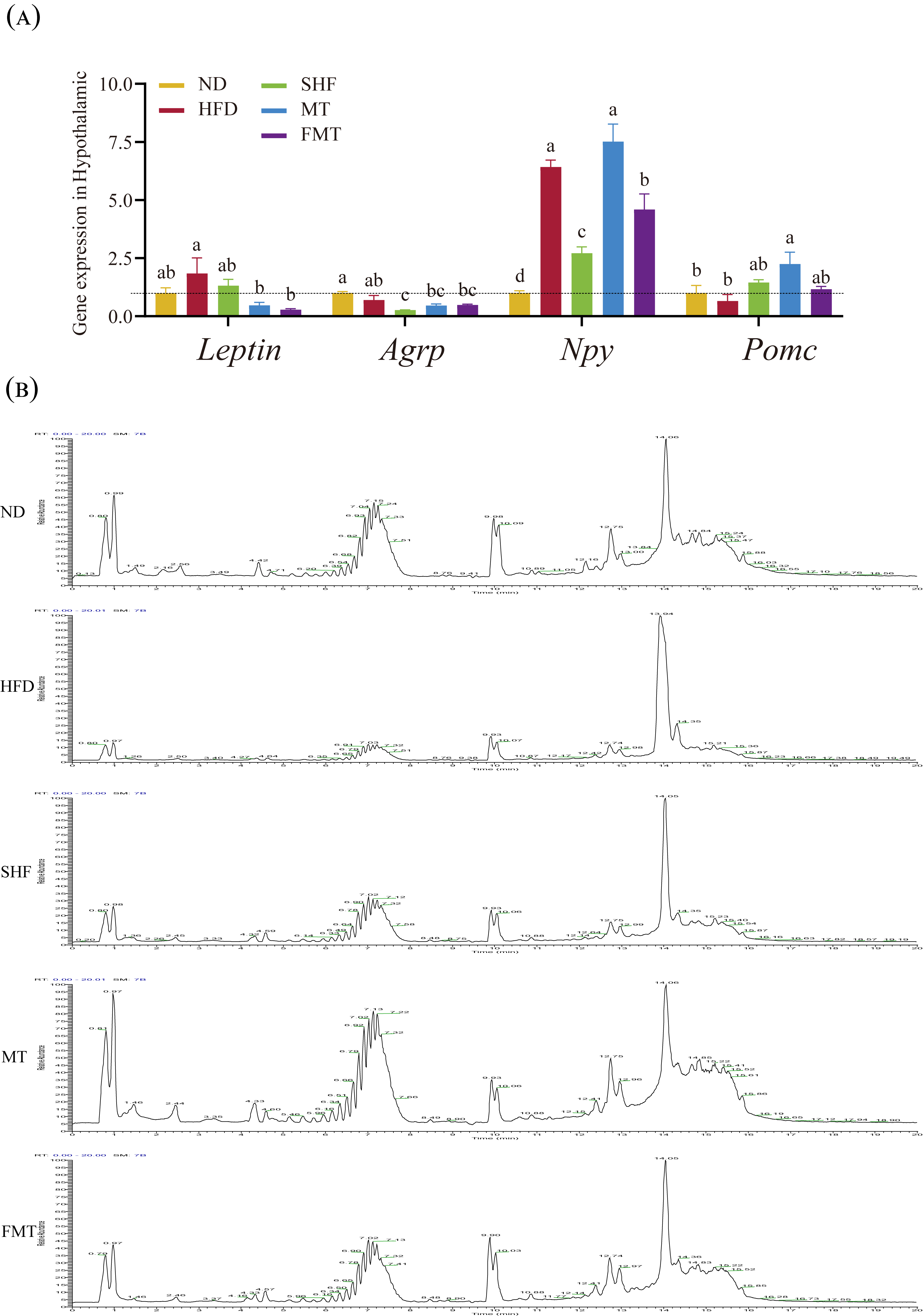
 **Supplementary Figure 2**

Partial gene expression (A) and QE/MS TIC plots (B) in the hypothalamus (n=4). Note: Significant differences are indicated by different letters on the bar graph (*p* < 0.05).


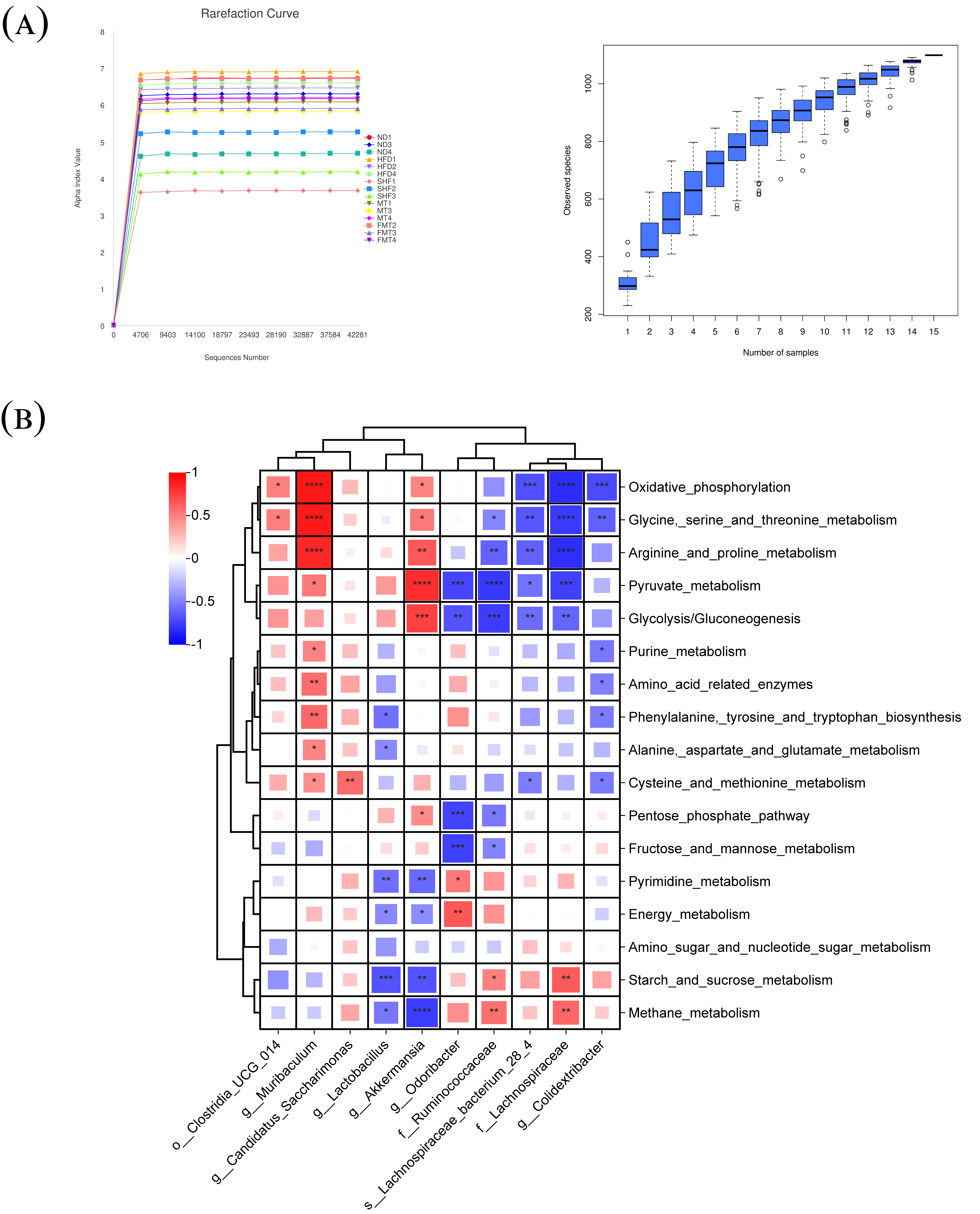
 **Supplementary Figure 3**

The species accumulation curve and dilution curve (A) and correlations between dominant bacteria in each group and certain predicted metabolic functions (B) (n=4). Note: **p* < 0.05, ***p* < 0.01, ****p* < 0.005, *****p* < 0.001

**Supplementary Table. 1 Metabolites information.**

| No. | Common Name | Chemical Formula | Average Molecular Weight | HMBD ID |
| --- | --- | --- | --- | --- |
|  | **Alkaloids and derivatives** | | | |
| 1 | Tropine | C_8_H_15_NO | 141.2108 | HMDB0259297 |
|  | **Benzenoids** | | | |
| 1 | 3,3'-Dimethoxybenzidine | C_14_H_16_N_2_O_2_ | 244.289 | HMDB0246003 |
| 2 | Benzophenone | C_13_H_10_O | 182.2179 | HMDB0032049 |
| 3 | Didecyl phthalate | C_28_H_46_O_4_ | 446.672 | HMDB0251217 |
| 4 | N,N-Dimethyl-p-toluidine | C_9_H_13_N | 135.21 | HMDB0255257 |
| 5 | Neostigmine | C_12_H_19_N_2_O_2_ | 223.2915 | HMDB0015472 |
| 6 | Hippuric acid | C_9_H_9_NO_3_ | 179.1727 | HMDB0000714 |
|  | **Lipids and lipid-like molecules** | | | |
| 1 | 1-Stearoylglycerol | C_21_H_42_O_4_ | 358.563 | HMDB0244009 |
| 2 | DL-Acetylcarnitine | C_9_H_17_NO_4_ | 203.2356 | HMDB0240773 |
| 3 | Docosapentaenoic acid (22n-6) | C_22_H_34_O_2_ | 330.5042 | HMDB0001976 |
| 4 | Arachidonic acid | C_20_H_32_O_2_ | 304.4669 | HMDB0001043 |
| 5 | Corticosterone | C_21_H_30_O_4_ | 346.4605 | HMDB0001547 |
| 6 | Sphingosine 1-phosphate | C_18_H_38_NO_5_P | 379.4718 | HMDB0000277 |
| 7 | Docosahexaenoic acid | C_22_H_32_O_2_ | 328.4883 | HMDB0002183 |
| 8 | Docosapentaenoic acid (22n-3) | C_22_H_34_O_2_ | 330.5042 | HMDB0006528 |
| 9 | Hexanoylcarnitine | C_13_H_25_NO_4_ | 260.3498 | HMDB0000756 |
| 10 | Oleamide | C_18_H_35_NO | 281.4766 | HMDB0002117 |
| 11 | Palmitoleic acid | C_16_H_30_O_2_ | 254.4082 | HMDB0003229 |
| 12 | SM(d18:1/16:0) | C_39_H_79_N_2_O_6_P | 703.0281 | HMDB0010169 |
| 13 | Palmitoylcarnitine | C_23_H_46_NO_4_ | 400.623 | HMDB0000222 |
| 14 | Pantothenol | C_9_H_19_NO_4_ | 205.2515 | HMDB0304820 |
| 15 | Taurocholic acid | C_26_H_45_NO_7_S | 515.703 | HMDB0000036 |
| 16 | Taurodeoxycholic acid | C_26_H_45_NO_6_S | 499.704 | HMDB0000896 |
| 17 | 13-Docosenamide | C_22_H_43_NO | 337.592 | HMDB0244507 |
| 18 | Propionylcarnitine | C_10_H_19_NO_4_ | 217.265 | HMDB0000824 |
|  | **Organic acids and derivatives** | | | |
| 1 | Betaine | C_5_H_12_NO_2_ | 118.1543 | HMDB0000043 |
| 2 | Capryloylglycine | C_10_H_19_NO_3_ | 201.2628 | HMDB0000832 |
| 3 | Creatine | C_4_H_9_N_3_O_2_ | 131.1332 | HMDB0000064 |
| 4 | DL-Arginine | C_6_H_14_N_4_O_2_ | 174.204 | HMDB0251511 |
| 5 | Glutamine | C_5_H_10_N_2_O_3_ | 146.1445 | HMDB0000641 |
| 6 | Lysine | C_6_H_14_N_2_O_2_ | 146.1876 | HMDB0000182 |
| 7 | Proline betaine | C_7_H_13_NO_2_ | 143.1836 | HMDB0004827 |
| 8 | DL-2-Aminooctanoic acid | C_8_H_17_NO_2_ | 159.2261 | HMDB0000991 |
| 9 | Isoleucine | C_6_H_13_NO_2_ | 131.1729 | HMDB0000172 |
| 10 | L-Pipecolic acid | C_6_H_11_NO_2_ | 129.157 | HMDB0000716 |
| 11 | Citrulline | C_6_H_13_N_3_O_3_ | 175.1857 | HMDB0000904 |
| 12 | Ornithine | C_5_H_12_N_2_O_2_ | 132.161 | HMDB0000214 |
| 13 | L-Alloisoleucine | C_6_H_13_NO_2_ | 131.1729 | HMDB0000557 |
| 14 | L-Norleucine | C_6_H_13_NO_2_ | 131.1729 | HMDB0001645 |
| 15 | Phenylalanine | C_9_H_11_NO_2_ | 165.1891 | HMDB0000159 |
| 16 | N6-Acetyl-L-lysine | C_8_H_16_N_2_O_3_ | 188.2242 | HMDB0000206 |
| 17 | Palmitoylethanolamide | C_18_H_37_NO_2_ | 299.4919 | HMDB0002100 |
| 18 | Pipecolic acid | C_6_H_11_NO_2_ | 129.157 | HMDB0000070 |
| 19 | L-prolyl-L-leucine | C_11_H_20_N_2_O_3_ | 228.292 | HMDB0253028 |
| 20 | Taurine | C_2_H_7_NO_3_S | 125.147 | HMDB0000251 |
| 21 | Valine | C_5_H_11_NO_2_ | 117.1463 | HMDB0000883 |
| 22 | gamma-Glutamylglutamic acid | C_10_H_16_N_2_O_7_ | 276.2432 | HMDB0011737 |
| 23 | DL-Glutamate | C_5_H_9_NO_4_ | 147.1293 | HMDB0060475 |
| 24 | Histidine | C_6_H_9_N_3_O_2_ | 155.1546 | HMDB0000177 |
| 25 | Pyroglutamic acid | C_5_H_7_NO_3_ | 129.114 | HMDB0000267 |
| 26 | L-Tyrosine | C_9_H_11_NO_3_ | 181.1885 | HMDB0000158 |
| 27 | N-Acetyl-DL-tryptophan | C_13_H_14_N_2_O_3_ | 246.266 | HMDB0255052 |
| 28 | N-Acetyl-Leu | C_8_H_15_NO_3_ | 173.2096 | HMDB0011756 |
| 29 | Valpromide | C_8_H_17_NO | 143.2267 | HMDB0259756 |
| 30 | Tetradecylamine | C_14_H_31_N | 213.409 | HMDB0258887 |
| 31 | Choline | C_5_H_14_NO | 104.1708 | HMDB0000097 |
| 32 | L-Carnitine | C_7_H_15_NO_3_ | 161.1989 | HMDB0000062 |
| 33 | Dodecylamine | C_12_H_27_N | 185.355 | HMDB0251571 |
| 34 | Linoleoyl Ethanolamide | C_20_H_37_NO_2_ | 323.5133 | HMDB0012252 |
| 35 | Oleoylethanolamide | C_20_H_39_NO_2_ | 325.5292 | HMDB0002088 |
| 36 | Stearoylethanolamide | C_20_H_41_NO_2_ | 327.545 | HMDB0013078 |
| 37 | Alpha-Linolenoyl ethanolamide | C_20_H_35_NO_2_ | 321.4974 | HMDB0013624 |
| 38 | Diethanolamine | C_4_H_11_NO_2_ | 105.1356 | HMDB0004437 |
| 39 | Sphingosine | C_18_H_37_NO_2_ | 299.4919 | HMDB0000252 |
| 40 | Kynurenine | C_10_H_12_N_2_O_3_ | 208.2139 | HMDB0000684 |
| 41 | Pentaethylene glycol | C_10_H_22_O_6_ | 238.2781 | HMDB0256256 |
| 42 | Hexaethylene glycol | C_12_H_26_O_7_ | 282.3306 | HMDB0061822 |
| 43 | Triethylene glycol monobutyl ether | C_10_H_22_O_4_ | 206.282 | HMDB0341407 |
|  | **Organoheterocyclic compounds** | | | |
| 1 | Tempo | C_9_H_18_NO | 156.249 | HMDB0258805 |
| 2 | 5-Hydroxyindoleacetic acid | C_10_H_9_NO_3_ | 191.1834 | HMDB0000763 |
| 3 | Cytosine | C_4_H_5_N_3_O | 111.102 | HMDB0000630 |
| 4 | Indirubin | C_16_H_10_N_2_O_2_ | 262.268 | HMDB0240743 |
| 5 | Indolelactic acid | C_11_H_11_NO_3_ | 205.2099 | HMDB0000671 |
| 6 | Indole-3-methyl acetate | C_11_H_11_NO_2_ | 189.2105 | HMDB0029738 |
| 7 | Miglitol | C_8_H_17_NO_5_ | 207.2243 | HMDB0014634 |
| 8 | Nicotinamide | C_6_H_6_N_2_O | 122.1246 | HMDB0001406 |
| 9 | Indoleacrylic acid | C_11_H_9_NO_2_ | 187.198 | HMDB0000734 |
| 10 | Nicotinic acid | C_6_H_5_NO_2_ | 123.1094 | HMDB0001488 |
| 11 | Serotonin | C_10_H_12_N_2_O | 176.2151 | HMDB0000259 |
| 12 | Uric acid | C_5_H_4_N_4_O_3_ | 168.1103 | HMDB0000289 |
|  | **Organosulfur compounds** | | | |
| 1 | beta-Mercaptoethanol | C_2_H_6_OS | 78.133 | HMDB0245190 |
|  | **Phenylpropanoids and polyketides** | | | |
| 1 | 4-Hydroxycinnamic acid | C_9_H_8_O_3_ | 164.158 | HMDB0002035 |
| 2 | 4-Dimethylaminocinnamaldehyde | C_11_H_13_NO | 175.231 | HMDB0247143 |
| 3 | Umbelliferone | C_9_H_6_O_3_ | 162.144 | HMDB0029865 |
